# Effects of public policy interventions for environmentally sustainable food consumption: a systematic map of available evidence

**DOI:** 10.1186/s13750-024-00333-6

**Published:** 2024-04-12

**Authors:** Ylva Ran, Pierre Van Rysselberge, Biljana Macura, U. Martin Persson, Assem Abu Hatab, Malin Jonell, Therese Lindahl, Elin Röös

**Affiliations:** 1 Department of Energy and Technology, Swedish University of Agricultural Sciences, 750 07 Uppsala, Sweden; 2 Stockholm Environment Institute, P.O. Box 24218, 104 51 Stockholm, Sweden; 3 Physical Resource Theory, Department of Space, Earth and Environment, Chalmers University of Technology, 412 96 Gothenburg, Sweden; 4 Nordic Africa Institute, Box 1703, 751 47 Uppsala, Sweden; 5 Department of Economics, Swedish University of Agricultural Sciences, 750 07 Uppsala, Sweden; 6 Beijer Institute of Ecological Economics, The Royal Swedish Academy of Sciences, 104 05 Stockholm, Sweden; 7 Stockholm Resilience Centre, Stockholm University, Stockholm, Sweden; 8 Global Economic Dynamics and the Biosphere, The Royal Swedish Academy of Sciences, Stockholm, Sweden

**Keywords:** Biodiversity loss, Climate change, Environmental impacts, Greenhouse gas emissions, Policy intervention, Sustainable consumption, Sustainable diets, Sustainable food systems, Demand-side interventions

## Abstract

**Background:**

The global food system is inflicting substantial environmental harm, necessitating a shift towards more environmentally sustainable food consumption practices. Policy interventions, for example, information campaigns, taxes and subsidies and changes in the choice context are essential to stimulate sustainable change, but their effectiveness in achieving environmental goals remains inadequately understood. Existing literature lacks a comprehensive synthesis of evidence on the role of public policies in promoting sustainable food consumption. Our systematic map addressed this gap by collecting and categorising research evidence on public policy interventions aimed at establishing environmentally sustainable food consumption patterns, in order to answer the primary research question: What evidence exists on the effects of public policy interventions for achieving environmentally sustainable food consumption?

**Methods:**

Searches for relevant records (in English) were performed in WoS, Scopus, ASSIA, ProQuest Dissertation and Theses, EconLit, Google Scholar and in bibliographies of relevant reviews. A grey literature search was also performed on 28 specialist websites (searches were made in the original language of the webpages and publications in English, Swedish, Danish and Norwegian were eligible) and Google Scholar (search in English). Screening was performed at title/abstract and full-text levels, with machine learning-aided priority screening at title/abstract level. Eligibility criteria encompassed settings, interventions (public policies on sustainable food consumption), target groups and outcomes. No critical appraisal of study validity was conducted. Data coding covered bibliographic details, study characteristics, intervention types and outcomes. Evidence was categorised into intervention types and subcategories. Visual representation utilised bar plots, diagrams, heatmaps and an evidence atlas. This produced a comprehensive overview of effects of public policy interventions on sustainable food consumption patterns.

**Review findings:**

The evidence base included 227 articles (267 interventions), with 92% of studies in high-income countries and only 4% in low-income countries. Quantitative studies dominated (83%), followed by mixed methods (16%) and qualitative studies (1%). Most interventions were information-based and 50% of reviewed studies looked at labels. Information campaigns/education interventions constituted 10% of the sample, and menu design changes and restriction/editing of choice context 8% each. Market-based interventions represented 13% of total interventions, of which two-thirds were taxes. Administrative interventions were rare (< 1%). Proxies for environmental impact (85%) were more frequent outcome measures than direct impacts (15%). Animal-source food consumption was commonly used (19%) for effects of interventions on, for example, greenhouse gas emissions. Most studies used stated preferences (61%) to evaluate interventions.

**Conclusions:**

The literature assessing policies for sustainable food consumption is dominated by studies on non-intrusive policy instruments; labels, information campaigns, menu design changes and editing choice contexts. There is a strong need for research on sustainable food policies to leave the lab and enter the real world, which will require support and cooperation of public and private sector stakeholders. Impact evaluations of large-scale interventions require scaling-up of available research funding and stronger multidisciplinary research, including collaborations with industry and other societal actors. Future research in this field should also go beyond the European and North American context, to obtain evidence on how to counteract increasing environmental pressures from food consumption worldwide.

**Supplementary Information:**

The online version contains supplementary material available at 10.1186/s13750-024-00333-6.

## Background

The global food system causes immense environmental impacts, e.g. it is responsible for more than one-third of greenhouse gas (GHG) emissions [1] and requires large amounts of water [2] and land [3, 4]. Agricultural land conversion is one of the largest causes of biodiversity loss [5]. Food production is driven by consumption of food, where growing global demand for food exacerbated by global population growth, urbanisation and increasing affluence, is expected to put even more intense pressure on the world’s ecosystems [4, 6]. To stop further erosion of global natural capital, a food system transformation is necessary, including drastic changes to food consumption patterns in affluent societies [7–9].

A transformation to a more sustainable food consumption requires targeted policy interventions to stimulate behaviour change along the value chain, including retailers and end-consumers [10, 11]. Public policies, i.e. actions taken by governments with specific goals and means [12–14] will be important in driving sustainable transformation of food systems [15, 16]. There is growing interest in policy interventions designed to mitigate environmental impacts of consumption, e.g. laws, taxes, labels and information campaigns [17–20]. However, there is little evidence on the effectiveness of such policy interventions and their impact on environmental outcomes [21–23].

Public policy interventions that can be used to transform food consumption to more sustainable forms include: (i) administrative policy interventions, such as laws, regulations and voluntary agreements; (ii) market-based policy interventions, such as taxes and subsidies; (iii) information-based policy interventions, such as labelling and provision of information; and (iv) behavioural policy interventions including choice editing and rationing [24].

Information-based interventions [24–26] aim to enable the “right” consumer choice by raising consumer knowledge, awareness and competence to choose sustainably [25, 27, 28]. They dominate the literature, most likely because they are relatively easy to implement compared with other interventions [26]. However, information-based interventions may fail to stimulate long-term sustainable change [27, 29].

Previous studies investigating public policies and policy interventions introduced by different levels of government to promote or incentivise sustainable food consumption are scattered across different sources. Moreover, existing literature on the role of public policies in influencing food consumption to achieve food system sustainability targets is insufficiently mapped. Numerous reviews have explored various measures and policies to promote dietary changes and reduce food waste, but these reviews have limitations in their scope and focus. Some concentrate exclusively on reducing consumption of animal-source foods [24, 30], while others examine interventions that aim to induce behaviour changes, without considering environmental impact [21, 22, 25, 31]. Some reviews focus solely on specific types of policies or outcomes, such as digital behavioural interventions [31], climate change mitigation [32, 33], food waste reduction [32] or health [20, 24, 34]. Further, previous systematic reviews and mapping studies do not explicitly address the role of government in promoting sustainable food consumption [31, 32]. Previous reviews also do not apply systematic evidence synthesis methodology [11, 19, 21, 22, 35] or do not focus on the measured effect of policy interventions [26]. Consequently, there is pressing need for a comprehensive evidence synthesis that consolidates and systematically maps available evidence on potential and existing public policy interventions targeting the establishment of environmentally sustainable food consumption patterns. Such an evidence map is imperative to inform meaningful decision-making in the realm of public policy.

### Objective of systematic mapping

The primary and overarching objective of this systematic map was to collect and describe available research evidence on existing and potential public policy interventions that have been implemented or suggested for achieving more environmentally sustainable food consumption patterns.

The primary research question in this systematic map was:

- What evidence exists on the effects of public policy interventions for achieving environmentally sustainable food consumption?

*(Environmentally) sustainable consumption*, e.g. food consumption, was defined as the use of goods and services to respond to basic needs and improve quality of life, while minimising the use of natural resources, toxic materials and emissions of waste and pollutants over their life cycle [9].

The question elements are as follows:

- *Setting(s):* Any geographical or economic setting.
- *Intervention(s):* Public policy intervention(s) implemented by national, regional or sub-national governments (or suggested to be implemented by e.g. researchers) with the explicit aim of achieving more environmentally sustainable food consumption patterns. We applied the IEE definition of a (public) policy intervention as any course of action, programme or activity taken or mandated primarily by national (and subnational) actors [36].
- *Outcome(s):* Anticipated or actual change in any type of environmental outcome of food production, or proxies thereof. Such proxies include e.g. change in consumption of meat or other animal-source food, plant-based food, food with high deforestation risk, or environmentally certified products.

## Methods

This systematic map followed Collaboration for Environmental Evidence (CEE) Guidelines and Standards for Environmental Evidence Synthesis in Environmental Management [37] and complied with RepOrting standards for Systematic Evidence Syntheses (ROSES) (see Additional file 1). The review was conducted following an existing protocol [38], with some minor deviations.

### Deviations from the protocol

The deviations from the protocol were relatively minor and can be summarised as follows:

1. Google Scholar searches in Nordic languages were omitted. Instead, searches for grey literature were performed on relevant websites, e.g. of agricultural and food ministries in all Nordic countries.
2. Calls for evidence on LinkedIn, ResearchGate or other social networks were not issued as records were collected from our large and diverse stakeholder group
3. All the title and abstract records were screened manually and machine learning with supervised classification was not applied in this review
4. Eligibility criteria were slightly rephrased from the protocol, but the essence of the criteria remain the same, with one exception. We excluded relevant reviews as we were looking for primary studies to identify eligible interventions. However, reviews were excluded under a separate exclusion code, both at title/abstract and full text level, and these were later searched for additional relevant records to be screened.
5. We do not include a list of excluded articles on title and abstract level because we had a very large number of excluded studies at this level. Instead, we provide a summary of exclusion reasons and the level of inclusion at the level of title and abstract screening
6. We chose not to map the extracted metadata against sustainable development goals or Sweden's 16 environmental quality objectives. This decision was based on the fact that these relationships were not readily apparent in the metadata.
7. We did not collect metadata pertaining to level of the jurisdiction of policy/policy intervention levers, actors targeted, theoretical framework, facilitators and barriers related to achieving policy impact or conflicts/synergies/indirect effects. This was due to absence of such information in the reported data and/or lack of comparability across the evidence base, rendering it irrelevant for this review.

### Stakeholder engagement

Stakeholder engagement helped inform the mapping, in a three-step co-design process with continuous stakeholder input to the map and search string, as outlined in the protocol [38]. First, in an online stakeholder workshop, we collected feedback on the project scope and boundaries, search strategy and search string. The stakeholder workshop was held on 29 October 2021 and involved 16 stakeholders from different key organisations. Second, feedback on a draft version of the protocol and search string was collected via an online survey and open consultation process that lasted 4.5 weeks (12 January–14 February 2022). The survey was sent to a broad group of Swedish and international stakeholders, and we received 14 responses from researchers, policymakers, research funders and other actors along the food value chain. Third, in a second stakeholder workshop, held on 17 February 2023, preliminary mapping results were discussed with 16 stakeholders and provided input on how to format results to fit the needs of key stakeholder groups and ways to take the systematic mapping results further were obtained.

The organisations represented in the stakeholder workshops and distribution of stakeholders that provided feedback on the survey are specified in the protocol [38] and a summary of feedback received are presented in Additional File 2 in the map protocol [38].

### Search for articles

#### Bibliographic sources and search engine

We searched five bibliographic sources and one search engine (Web of Science (WoS), Scopus, Applied Social Sciences Index & Abstracts (ASSIA), ProQuest Dissertation and Theses, EconLit and, Google Scholar) for relevant articles between 10 and 22 February 2022, as described in Table 1. Sources behind paywalls were accessed through subscriptions from the Swedish University of Agricultural Sciences (SLU). The searches were conducted using English language terms. Table 1 includes an example of search substrings used in Web of Science Core Collections. The search string was divided into three parts, based on search terms representing (A) food consumption, (B) interventions and (C) environmental sustainability. Each search string was developed in iterative cycles, based on expert inputs from the research team, the reference group, the stakeholder workshops and the SLU Library helpdesk function for Systematic reviews (SLU hub for systematic reviews) Search string development was described in the protocol [38].

**Table 1:** Search substrings (shown as formatted for Web of Science)

A	(((food OR meal* OR diet OR eating) NEAR/2 (purchas* OR select* OR choice* OR reduc* OR choose OR decision OR consum* OR intake OR behav* OR habit*)) OR "product select*" OR "food products" OR menu OR "food environment" OR "dietary pattern*" OR catering OR ((beverage* OR grocery OR groceries OR fish OR seafood OR beef OR meat OR dairy OR milk OR vegetable* OR legume* OR “meat alternative” or “organic food” or “local food”) NEAR/2 (consum* OR choice* OR choose OR select* OR market OR demand* OR reduc*)))
B	(policy OR policies OR legislat* OR law* OR ((label* OR labl* OR certifi*) NEAR/2 (food OR ecol* OR sustainab* OR carbon OR climate)) OR ecolabl* OR ecolabel* OR eco-labl* OR eco-label* OR eco-certif* OR guideline* OR guidance OR incenti* OR intervent* OR nudg* OR subsid OR stimul* OR persua* OR “voluntary agreement*” OR roundtable* OR forc* OR innovat* OR directive* OR regulation OR regulations OR education OR ((plate* or serving-size or "serving size") NEAR/1 ration*) OR ((carbon OR consum* OR output OR environmental) NEAR/2 (tax* OR information OR standard* OR ban* OR prohibit* OR limit* OR sanction*)) OR "green criteria" OR "public procurement" OR "green public procurement")
C	("climate change" OR "climatic change" OR "global warming" OR "greenhouse gases" OR ghg OR "greenhouse effect" OR "greenhouse gas" OR "carbon emission*" OR "carbon footprint" OR "water footprint" OR “land use” OR “biodiver*loss*” OR ecosystem OR overfishing OR pollution OR "over fishing" OR deforest* OR (reduction* NEAR/2 emission*) OR (environment* NEAR/2 (impact* OR consequence* OR assess* OR evaluat* OR indicator* OR mitigat*)) OR "plant based food" OR "plant-based food" OR “planetary health diet” OR plant-forward OR "pro-environmental" OR "local food" OR "seasonal food" OR "eat less" OR flexitarian OR vegan OR vegetarian OR pescetarian OR "meat reduction" OR "beef reduction" OR (sustainab* NEAR/2 (consum* OR diet* OR food OR fisher*)))

Each search source required specific adjustments of the string. Additional file 2.1 includes search details with the specific settings applied for each source (as per search functionalities of each source). For Google Scholar, we used simplified search strings. The first 1000 search results were extracted as citations using Publish or Perish software [39]. However, it's noteworthy that our search string exceeded the 256-character limit which appears to be imposed by Google Scholar. Consequently, we present the strings conforming to this limitation in Additional file 2.

#### Specialist websites

We searched for grey literature between 20 and 25 February 2023, on 28 different specialist websites (as listed in Additional file 2.2). The search was performed manually on each website (and its publications section if available). We used a set of basic search terms where websites allowed a keyword search. Basic search terms for all languages are listed in Additional file 2.3 and search results in Additional file 2.4a–c. Searches were performed in English, Swedish, Norwegian and Danish, as predefined by the language skills across the research group. The list of the relevant websites was compiled with inputs from stakeholders, as outlined above in the section ‘Stakeholder engagement’, and they included key governmental agencies for agriculture and food in all Nordic countries as well as relevant, international, non-governmental organisations in the area of food and sustainable food consumption.

#### Additional searches

We searched for relevant records in the bibliographies of 89 relevant reviews that were excluded during screening (26 at title and abstract level and 63 at full text level). Additional records were also supplemented by our stakeholders.

#### Search comprehensiveness

Consistency checking of the search string was performed through expert consultation with two librarians at the Swedish University of Agricultural Sciences and through a benchmark list of 38 articles developed by the whole research team (see the study protocol [38]), as described in the protocol. The final search string captured 36 of the 38 selected benchmark studies. The remaining two articles were not captured because they did not include mentions of food (consumption)-related terms in their titles, abstracts, or keywords. We attempted to include additional search terms to capture the remaining benchmark studies but these generated too much noise.

#### Assembling a library of search results

Results of the bibliographic searches were combined, and duplicates were removed prior to screening. A library of search results was assembled in EPPI-Reviewer.

### Article screening and study eligibility criteria

#### Screening process

**Fig. 1:**
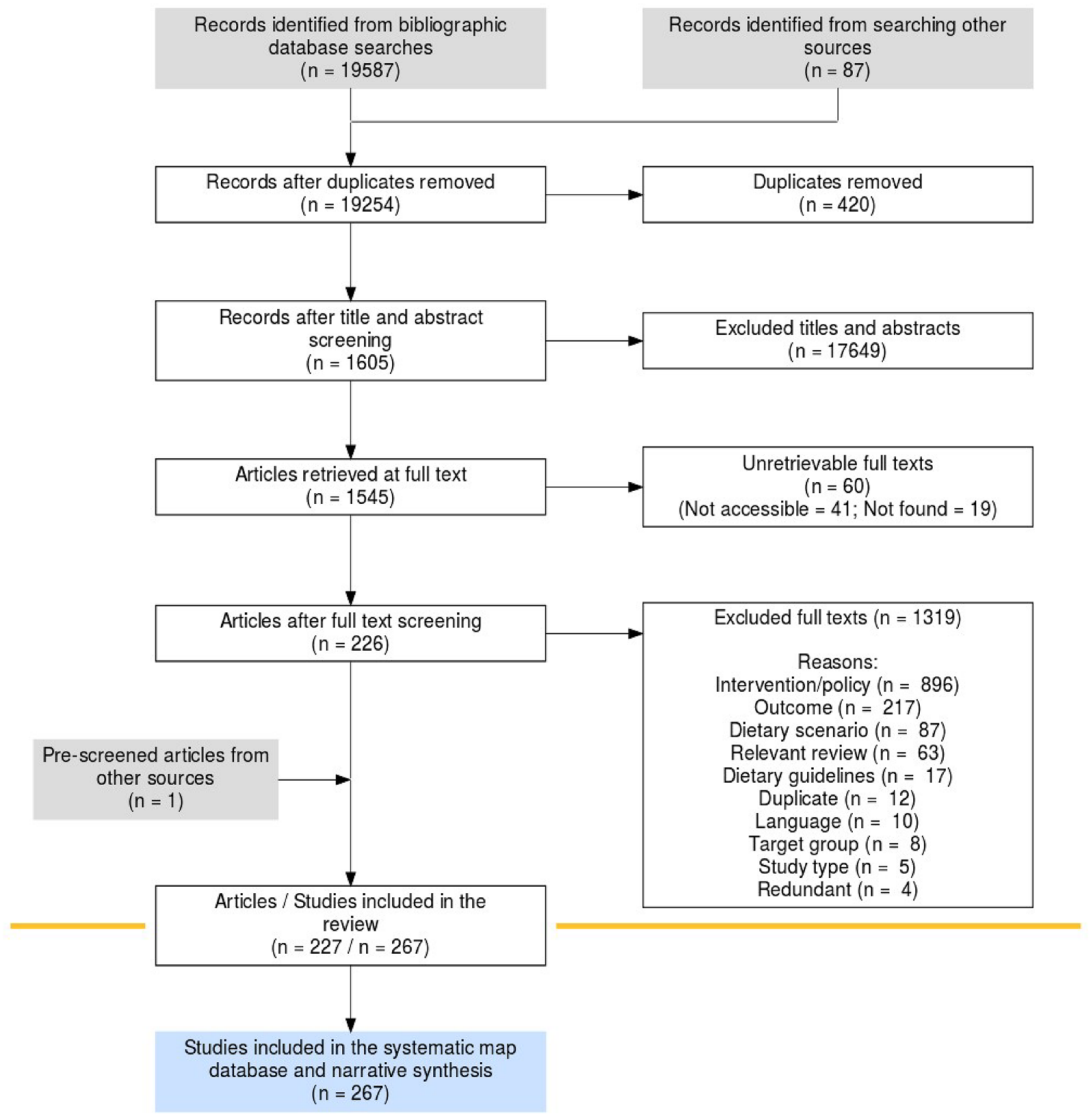
ROSES diagram illustrating the systematic mapping process. The diagram is developed using the ROSES flow diagram template for systematic maps Version 1.0 [41].

Records from bibliographic databases and Google Scholar were screened at two different levels: title and abstract, and full text. Records identified via searching bibliographies of relevant reviews were screened at both title and abstract and full text and records identified via expert consultation were screened at full text only (Fig. 1). In cases where abstracts and titles provided insufficient information, we included the records for full text screening.

At the title and abstract level, three reviewers (YR, ER, PvR) screened a subset of 474/19,254 records for consistency checking, reaching an agreement level of 87% after three screening rounds. All disagreements were discussed and resolved. The remaining records were screened by a single reviewer and equally shared between YR and PvR.

To increase screening efficiency, we used the priority screening functionality available in EPPI-Reviewer Web. Specifically, we used active learning-powered priority screening to support prioritisation of records for manual screening. This priority screening function adds relevant records to the beginning of the screening queue and moves less relevant records towards the end. Priority screening was used after consistency checking. All records were screened manually and by a human, and priority screening was not used to provide screening stopping criteria.

For screening at full text level, we started with consistency checking on a sample of 110/1545 full texts (screened by PvR and YR). An agreement level of 86% was reached after three screening rounds. All disagreements were discussed and resolved. The remaining articles were then screened separately by each reviewer, divided between PvR and YR. No records authored by the review team members were screened or coded by them.

Excluded articles at full text with reasons for exclusion are listed in Additional file 3.1. A list of records that could not be retrieved at full text is provided in Additional file 3.2.

#### Eligibility criteria

We applied the following eligibility criteria, as in the protocol [38]:

- *Eligible settings:* Private and public food consumption in any geographical or economic setting.
- *Eligible interventions:* Public policy interventions with the explicit aim of achieving more environmentally sustainable food consumption patterns (implemented by national or sub-national governments, or suggested for implementation). These included policy interventions aimed at achieving change in consumption of, e.g. meat, plant-based foods, deforestation-prone foods or certified products. The relevant intervention had the end-goal of directly or indirectly influencing consumer choice towards more sustainable foods. Production-side interventions, such as agricultural subsidies to promote more sustainable farming or interventions to restrict energy use in food production, were not included. Similarly, certification schemes aimed primarily at changing production, and not primarily at promoting more sustainable consumer choice, were not included. Policy interventions that aimed at reduction of overconsumption (food eaten in excess or food wasted) were also not considered in this review.
- *Eligible policy/policy intervention scales:* Local (universities, schools, hospitals and similar institutions), municipal, regional or national level.
- *Eligible target group of the policy/policy intervention:* Food service sector, including consumers, retailers, food industry (marketing, food reformulation), restaurants, public procurement, cooperatives, supermarkets and similar. Policy interventions regulating the production side (e.g., regulation of pesticide use) or directed at primary producers were not included.
- *Eligible outcomes:* Anticipated or actual change in any type of environmental impact of food production or proxies thereof, including, e.g., change in consumption of meat, plant-based foods, deforestation-prone foods, or certified products. Studies that measured and/or discussed health outcomes only were not included, although the interventions included in such studies, e.g., reduced overconsumption or consumption of red meat, might lead to environmental gains. Studies not explicitly measuring environmental outcomes (directly or indirectly) were not included.
- *Eligible study designs:* Modelling, observational and experimental studies, theoretical and conceptual studies, studies with quantitative, mixed method and qualitative data. Only primary research was included. Review articles were excluded, but their bibliographies were searched for relevant primary study records.
- *Eligible languages:* English, Swedish, Danish, and Norwegian (matching the skillset of the review team).
- *Time frame:* No limitations.

#### Study validity assessment

We did not conduct any study validity assessment as part of our systematic mapping, but we extracted metadata on study design (i.e. experimental/evaluation design, sample collection method, study location, preference) that can be used and expanded in critical appraisal in any future systematic reviews based on this map. As the eligible outcomes in this study are measures of change, the presence of a comparator is automatic. We have therefore also extracted metadata on type of comparator and comparator details (no intervention, alternative intervention, etc.

#### Data coding strategy

Following full-text screening, data from full texts were coded, including: (i) bibliographic information; (ii) research inquiry (for primary research): qualitative, quantitative, mixed; (iii) study type (including observation, experiment and model type), (iv) experimental/evaluation design (including type of comparator and comparator details (no intervention, alternative intervention, etc.,)) (v) bias towards age and gender, (vi) sample collection method and type of food consumption preference, (vii) study location; (viii) policy intervention type; and, (ix) outcomes measured and targeted, including (proxies for) environmental impacts. In regard to food consumption preference we distinguish between: stated preference, a hypothetically estimated preference illustrated by a stated choice; real revealed preference, which is based on established and actual records of behaviour and; modelled revealed preference, which is based on a modelled estimate of revealed behaviour [40].

A study in this systematic mapping exercise refers to a study of an intervention and an article can contain several studies. Different types of label designs for environmentally sustainable food consumption were considered as different levels of the same intervention in this mapping review, not as different interventions. A case refers to those instances where a study includes several types of outcomes, observations, experiments, models, label types, sample collection methods or is tested in several geographic locations and, thus, within one study there can be several cases. A code book is included in Additional File 4.

When required information was not stated in the text, it was marked “not stated”, alternatively we used “not applicable” for cases where the meta data code did not apply to a specific study. Data coding variables were iteratively developed by consulting the expertise within the research team, and then further advanced during the consistency checking phase.

To ensure consistency at the stage of metadata extraction, a subset of 20/227 records was coded by both YR and PvR. Disagreements were discussed until consensus was reached and clarifications and guidance on data codes were made in the discussions. When consensus on metadata coding was reached, metadata extraction was finalised, with PvR and YR coding equal shares of the included articles.

The evidence base was collated in a CSV file (Additional file 5). Each line in the database represents an intervention, i.e. a study, and each article can have several interventions.

#### Data mapping method

To identify knowledge clusters and gaps, interventions were first sorted inductively according to emerging themes and thereafter deductively, according to intervention categories from the literature (e.g. Reisch et al., 2013; Temme et al., 2020) as: (i) administrative policy interventions such as laws and regulations; (ii) market-based policy interventions such as taxes and subsidies; (iii) information-based policy interventions such as labelling and provision of environmental information; and (iv) behavioural policy interventions, including choice editing and rationing (Temme et al., 2020). There is an overlap between information-based interventions and some behavioural policy interventions and a clear distinction between these categories is difficult to make. We used heatmaps of the types of interventions across study types, experiment types, and, outcomes to identify knowledge gaps and clusters.

The metadata were visualised using bar plots, diagrams and tree maps produced in Microsoft Excel. Heatmaps (produced using ggplot 2 package in RStudio version 2023.03.0.) were used to show knowledge clusters and gaps. A choropleth map was produced with ArcGIS pro 3.1.1. The results were also visualised via an evidence atlas, to show the geographical spread of the evidence base. This atlas is available online.

## Review findings

### Searches and screening

The flow of information obtained in the review is illustrated in a ROSES flow diagram in Fig. 1. The initial searches yielded a total of 28,104 records from the bibliographic databases Web of Science Core collection, Scopus, ProQuest Dissertation and Theses, Econlit, Applied Social Sciences Index & Abstracts, and Google Scholar. We removed duplicates in two stages, first in EndNote software and then in EPPI reviewer. The searches for grey literature on specialist websites did not yield any additional eligible records for inclusion. We also searched the bibliographies of 89 relevant reviews, which generated an additional 87 records that , resulting in a total of 19,254 records screened at title and abstract level. The main reasons for exclusion at title and abstract level were; no eligible intervention (90% of records) or no relevant outcome (2% of records). We included 8% of the records at the level of title and abstract screening. A total of 60 full texts could not be retrieved (Additional file 3.2). We retrieved a total of 1545 records at full text level. Screening on full text resulted in 226 eligible articles for metadata extraction. Expert consultation yielded one additional full text record. Overall, our evidence base included 227 articles, reporting results for 267 individual studies.

### Publication year and geographical representativeness

**Fig. 2:**
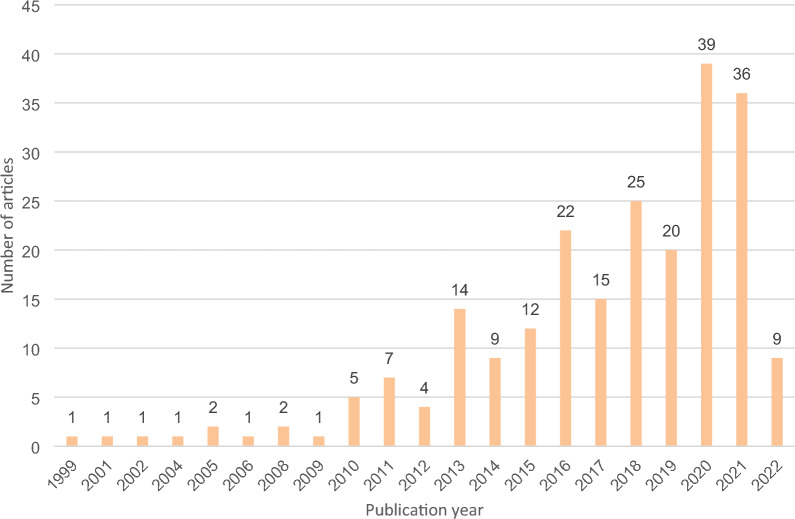
Number of articles included in mapping per year of publication, 1999–2022 (the search was completed in February 2022 and only included articles published before that point)

As illustrated in Fig. 2, few articles were published before 2011 (15, N = 227) but there was an increasing trend in publications after 2013, with 201 of the 227 articles in the evidence base (88%) published from 2013 onwards. There was also a clear increase in the number of articles per year over time, with the last two full years (2020–2021) comprising more than one-third of the total number of articles included in the analysis.

**Fig. 3:**
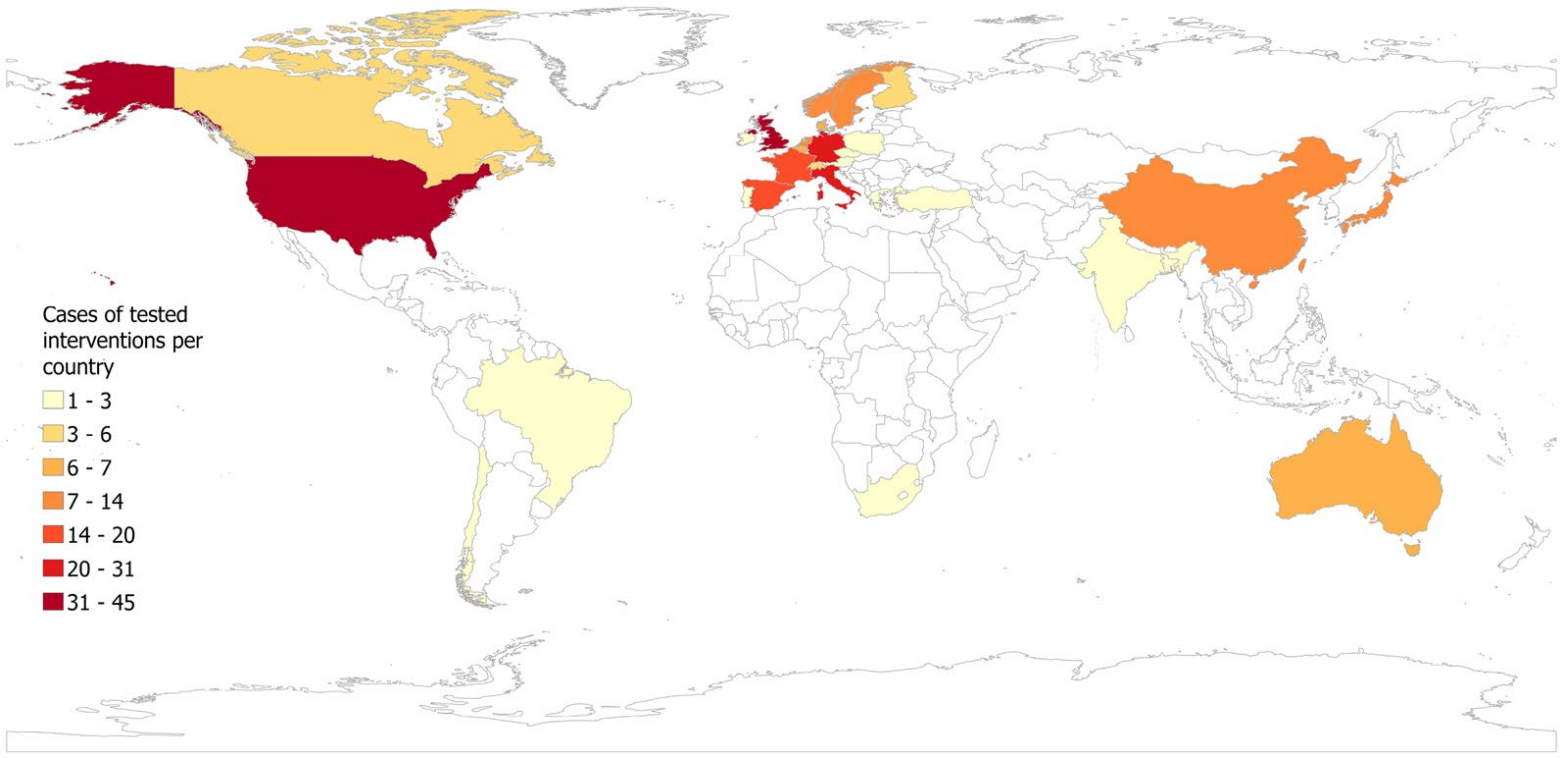
Frequency of cases where an intervention included in the evidence base was tested in a specific geographic location. Intensity of colour indicates number of cases per country

The vast majority of the cases included in the mapping were located in high-income countries. High-income countries have a gross national income of $13,846 or more and low-income countries have a gross national income of $1,135 or less [42]. In 285 cases where an intervention was tested in a specific geographic location 96%, N = 298 as several studies had multiple geographical locations) the test was located in a high-income country and only 4% in lower income countries (13, N = 298) (Fig. 3). Eight cases (3%, N = 298) had a global or regional focus and three (1%, N = 298) did not specify a geographical location. Nineteen cases (6%, N = 298) compared different countries and were conducted in several locations. The majority of intervention tests were located in the United States (45 cases, 15%, N = 298), followed by the United Kingdom (41 cases, 14%, N = 298) and Germany (31 cases, 10%, N = 298).

### Study type, evaluation design and comparator

Most of the studies were quantitative (84%, N = 267), followed by mixed-methods studies (41 studies, 16%) and only three studies were qualitative (1%). As shown in Table 2, most studies used a combination of experimental and observational methods (165 studies, 62%, N = 267) followed by experimental (46 studies, 17%) and modelling (41 studies, 15%). Table 2 also shows the most common evaluation design for each study type. Note that several observation, experiment, and model evaluation types can be used in a study. For observation and experiment studies, most used survey-based choice experiments (75 studies), often accompanied by additional observations collected via surveys. Many observation and experiment studies also used nudge experiments/information intervention experiments (64 studies), often combined with a survey or focus group discussions to collect data.

**Table 2:** Number of cases for each observation type, model type and experiment type across study types (N = 267)^a^

Study type	Observation type (Number of cases)			Experiment type (Number of cases)					Model type (Number of cases)		
	Survey	Interview	FGD	Survey-based choice experiment	Choice experiment	RCT	Economic experiment	Nudge/information intervention experiment	Simul-ation model	Optimi-sation model	Other model
Experiment (49 studies)				6	7	7	9	20			
Experiment and model (4 studies)							2	2		4	
Model (41 studies)									35	5	1
Observation (7 studies)	6	1	1								
Observation and experiment (165 studies)	157	15	26	75	7	3	21	58			
Observation and model (1 study)	1	1									1

Key: Focus Group Discussion (FGD), Randomised control trial (RCT).

^a^Note that one study can include several cases of observations, experiments and/or modelling types.

The second largest study type was experimental studies (49 studies, 18%, N = 267) and these were most often evaluated using nudge experiments/information intervention experiments [21], compared with cases using choice experiments. Observation studies constituted a smaller sample (7 studies, 3%, N = 267) and were mainly performed using surveys. Modelling was used in 41 studies (15%, N = 267) and 85% of these used simulation models (35 studies).

Random experimental design with random allocation to groups was applied in 115 of studies (43%, N = 267) followed by one group post-test in 35 studies (13%) and one group pre-post-test in 19 studies (7%). Experiments with non-random allocation to groups were used in 30 studies (11%, N = 267). Less common study designs were quantitative and qualitative descriptions, where no specific methodology was clearly stated, which were used in three and two studies respectively (≤ 1%, N = 267) and case studies, which were used in three studies (1%).

**Fig. 4:**
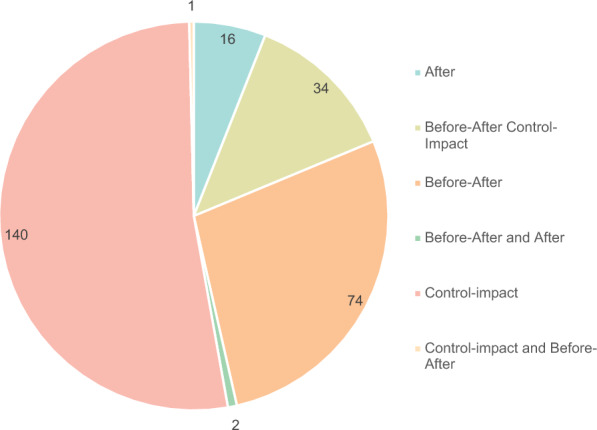
Type of comparator used in the different studies in the evidence base (N = 267)

The most common type of comparison was control-impact, (140 studies, 52%, N = 267), followed by before and after in 74 studies (28%) (Fig. 4). Only 13% of studies had both spatial and temporal comparators (before-after-control-impact). Three studies used more than one comparator.

Additionally, interventions were evaluated either by comparing the intervention to no intervention (110 studies, 41%, N = 267), followed by intervention compared to no intervention and an alternative intervention in 68 studies (22%). In 48 studies (18%, N = 267), the intervention was compared to no intervention and a different level of intervention (Table 3). Different levels of the same intervention were used as the only comparator in 8% of the studies (Table 3).

**Table 3:** Comparator details for each study in the evidence base (N = 267)

Comparator details	No. of studies
No intervention	1
No intervention and alternative intervention	68
No intervention and different level of intervention	51
Different level of intervention	18
Alternative intervention	13
Different level of intervention and alternative intervention	3
No intervention, different level of intervention, and alternative intervention	9

#### Sample collection method and gender and age bias

Sample populations used for evaluation of interventions were most often recruited by convenience sampling with no panel (128 cases, 57%, N = 225 as information on sample population collection was not applicable in 50 studies and some studies used several sample collection methods). This was followed by convenience sampling with a panel, used in 35 cases (17%). In the cases where a panel was used for recruiting subjects (N = 67), for around half (33 cases) the nature of the panel was unspecified, while self-recruited web panels were used in 20 of the cases, randomly selected web-panels in nine cases and other type of randomly selected panels in five cases. Twelve cases in total used quota sampling (5%, N = 225), while for 14 cases (6%) the sampling collection method was not stated at all.

Of the studies that presented sample data regarding age (N = 188), 63% either showed no bias or did not clearly specify a bias towards a specific age group (119 studies), 10% presented a bias where the sample population had a higher mean age than the reference population (18 studies) and 28% presented a bias where the sample population had a lower mean age than the reference population (52 studies). Of studies that presented sample population data regarding gender (N = 188), 51% presented a bias towards women in their sample population (96), 7% a bias toward men (14 studies) and 38% showed no bias towards gender or such bias was unclearly presented (77 studies). In 31 studies for age and 32 studies for gender, sample data in regard to age or gender were not presented.

#### Type of intervention

**Fig. 5:**
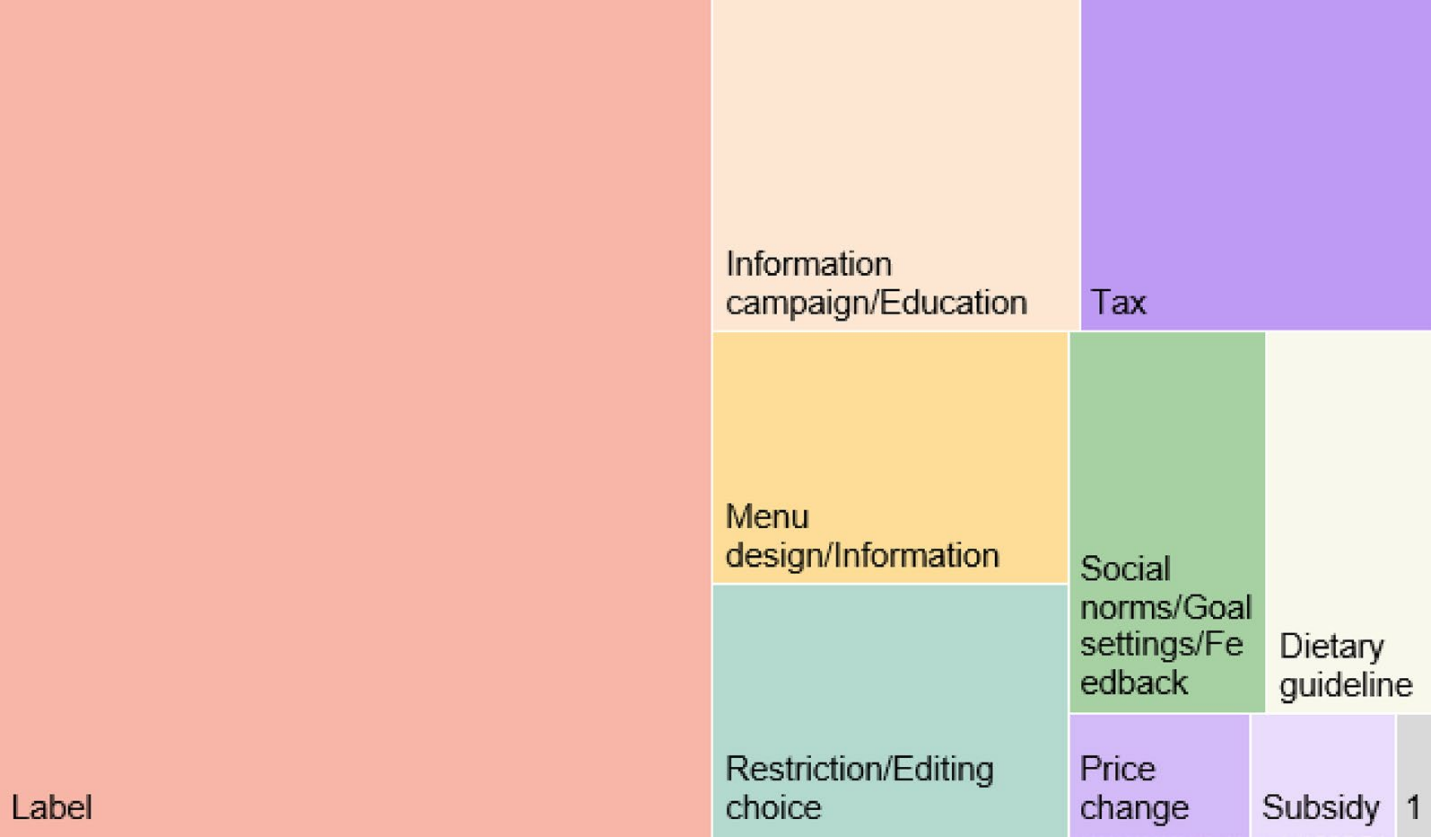
Tree map showing type of interventions categorised in emerging clusters in the evidence base. Note that ‘Price change’ indicates studies on a change in price for a food/menu option where it was not stated if the price change would be implemented as, e.g., a tax or a subsidy. Key: 1 = Laws

We identified 267 studies of interventions and categorised them (Fig. 5). The majority of interventions included in the evidence base were information-based. A majority of studies (133) evaluated effects of labels or front-of-package environmental information (50%, N = 267). In cases where labels were studied, 59 tested more than one type of environmental label resulting in 192 labels tested. The majority of cases tested eco-labels, including labels for organic production (28%, N = 192), followed by different types of carbon labels (24%), other types of labels, e.g. sustainability labels or “environmentally friendly food” labels (21%), and labelling on food origin, including locally produced foods (16%). In addition, 10% of the cases tested other types of front-of-package information that was not directly a label, such as information about a food environmental impact, provided in a choice experiment.

Other types of information-based interventions that were tested more seldom were different types of information-based campaigns and education on more environmentally friendly food consumption, including a combination of different interventions, which were evaluated in 27 studies (10%, N = 267). Information provided on menus, e.g. leaves indicating “green choices,” or changes in menu design to promote more sustainable choices, constituted 8% of the total sample (20, N = 267). An additional 14 studies (5%, N = 267) evaluated different types of dietary guidelines based on e.g. the EAT-*Lancet* Commission’s reference diet [7] or Eat Well diets [43]. These studies primarily modelled the environmental outcome assuming that a population within a specific geographical area would follow these new recommendations. A few studies amongst these evaluated dietary guidelines for public procurement (4 studies) and only one out of the total 14 investigated actual compliance with dietary guidelines [44].

For interventions that focus on behavioural aspects, restriction or editing of the choice context, e.g. changing the order of foods/meals in a counter, default nudges or removing or replacing certain options on a menu, were tested in 20 studies (8%, N = 267). Market-based interventions were evaluated in 13% of the studies (35, N = 267) and of these, taxes were the most common intervention (26 studies, 74% of market-based interventions, N = 35), followed by a change in price, but where it was not specified whether the change was a result of a tax or a subsidy (5 studies, 14% of market based interventions). Subsidies were evaluated in four studies (11% of market-based interventions). One intervention (< 1%, N = 267) tested an administrative intervention where a theoretical law about how public procurement must reduce meat consumption, and the effects of launching such a law on consumer behaviour, was evaluated in regard to food shopping habits.

**Fig. 6:**
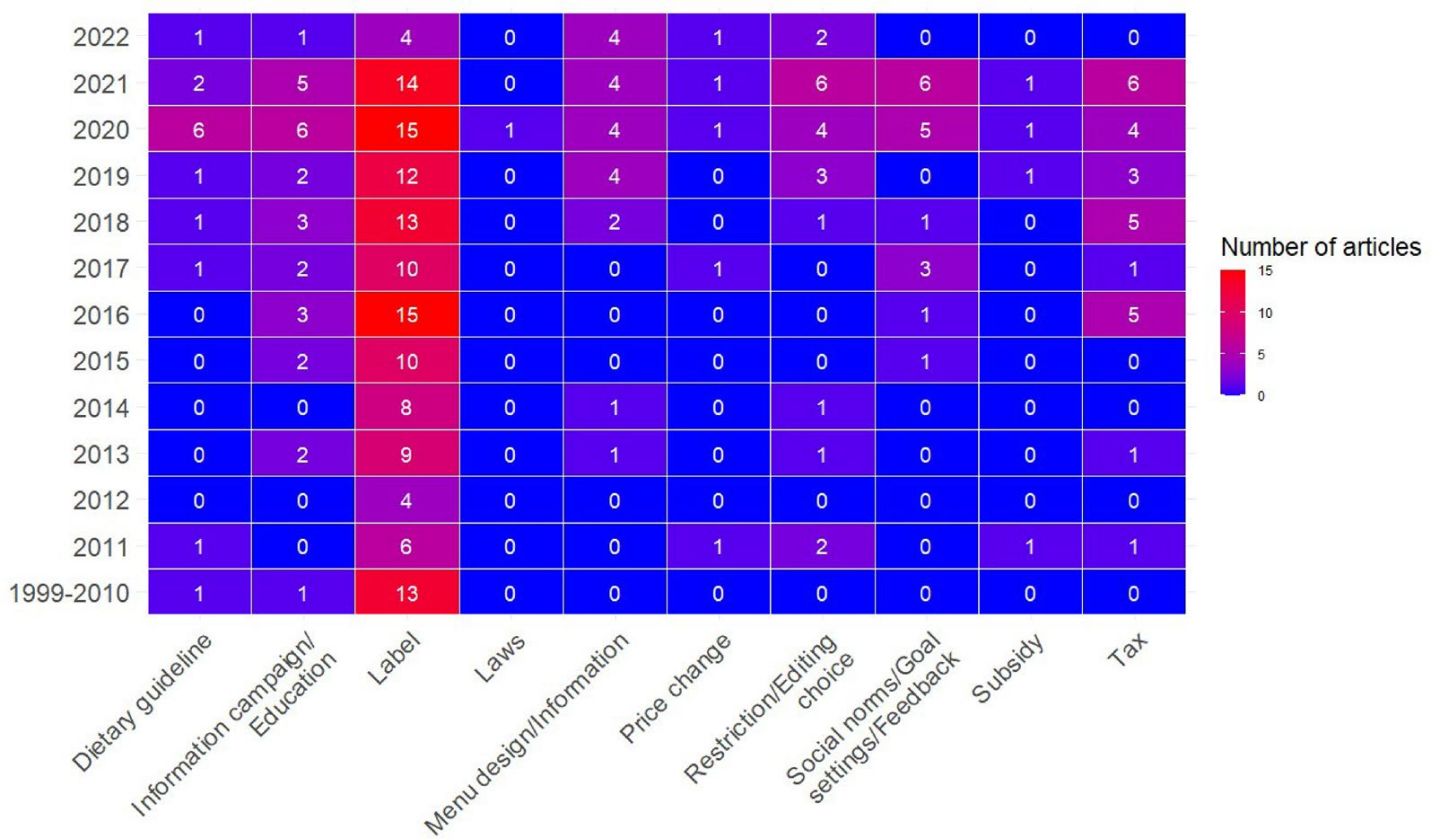
Number of different intervention subcategories in articles in the evidence base over time, 1999–2022

Mapping the number of articles for different types of intervention subcategories over time revealed an overall increasing trend over time, particularly from 2016 onwards (Fig. 6). There were clear peaks in the period 2019–2021 in the number of studies evaluating taxes, information campaigns/education, restriction/editing choice and dietary guidelines. The number of studies on labels also increased over time, but the relative increase over time was smaller than for the other subcategories.

#### Stated or real preference

**Fig. 7:**
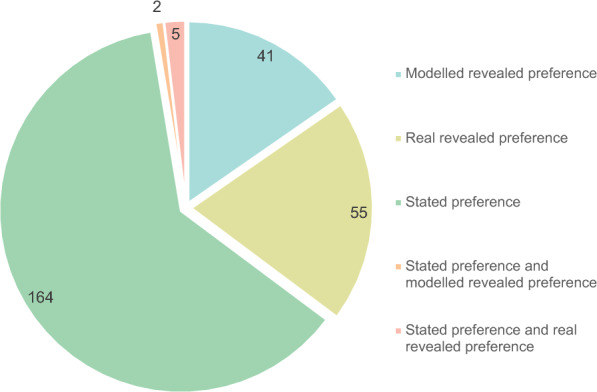
Number of interventions in the evidence base evaluated by stated preference, real revealed preference or modelled revealed preference

We also investigated whether interventions were evaluated with stated, real revealed, or modelled revealed preferences (Fig. 7). The majority of interventions (164 studies) were evaluated using stated preferences, (61%, N = 267) compared with 21% evaluated by real revealed preferences and about 15% with modelled revealed preferences. An additional five interventions or 2% were tested using both stated and real revealed preferences, while two interventions were tested with modelled *and* stated preference.

#### Measured outcomes

**Fig. 8:**
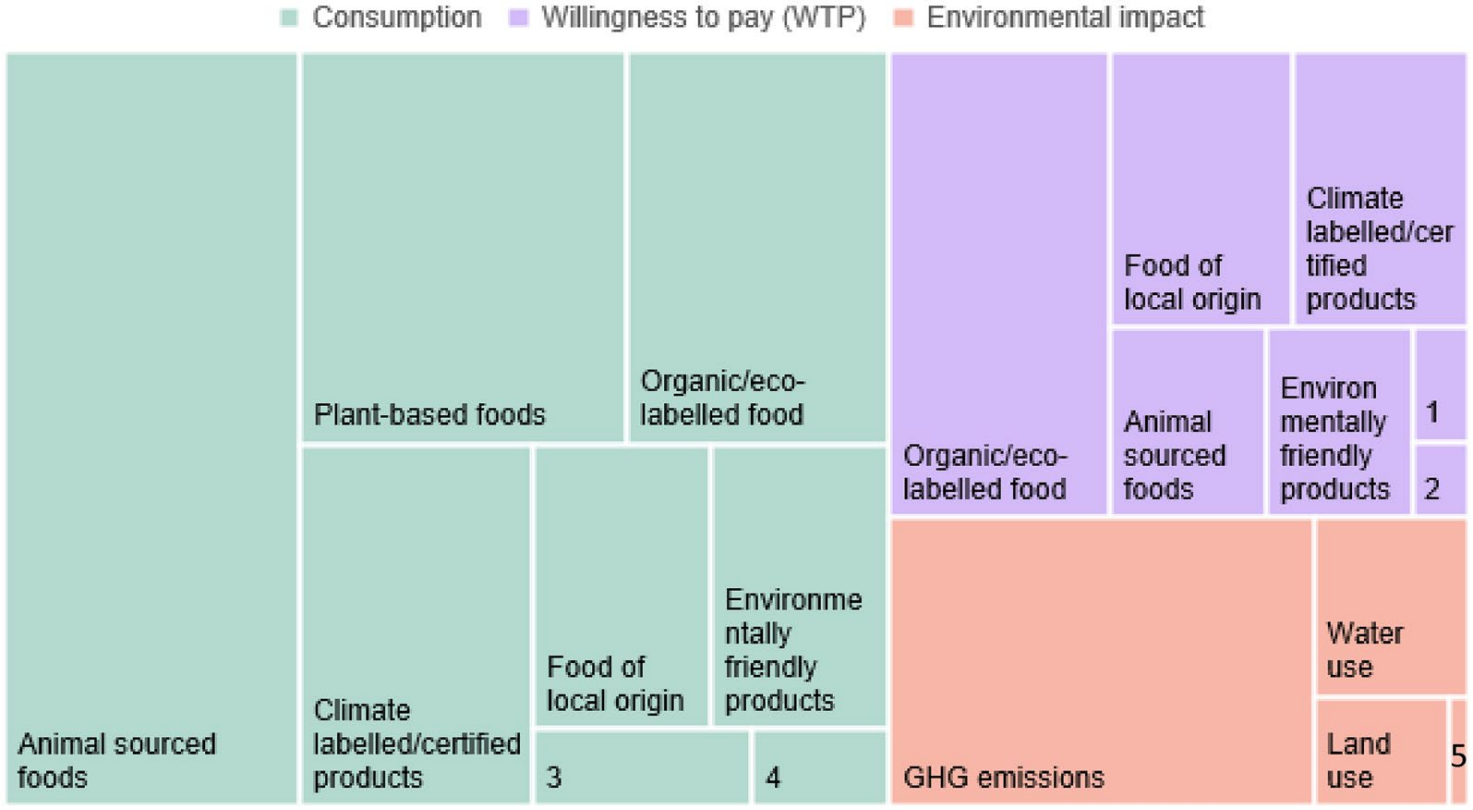
Tree map showing the relative distribution of interventions with different types of environmental outcomes. Note that a study can measure several outcomes. Key: 1. Plant-based food. 2. Deforestation-prone/certified food. 3. Food with less environmental impact. 4. Deforestation-prone/certified food. 5. Energy use.

The outcomes measured for each intervention varied widely between studies and many studies also evaluated several outcomes for the same intervention. Figure 8 illustrates the relative distribution of evaluated outcomes for all studies, categorised into outcomes considering (i) direct environmental impact or effect, such as GHG emissions and water use, (ii) proxies for environmental impact as [changes in] consumption of different foods, e.g. meat consumption, and (iii) proxies for environmental impact as [changes in] willingness to pay (WTP) for different foods (indicating consumption changes), such as carbon-labelled food or sustainable meat products.

As Fig. 8 shows, proxies for environmental impact were evaluated more often than direct environmental impact. Note that one study can measure several outcomes. In the evidence base, proxies constituted 81% of the cases of evaluated outcomes (446, N = 547, compared with 19% of evaluated outcomes corresponding to direct environmental effects (101, N = 547).

Consumption of animal-source foods was the proxy most commonly measured, and also the most evaluated outcome for all interventions, 19% of total measured outcomes (105, N = 547). Other proxies that were often measured were consumption of plant-based food (63, 11%, N = 547), organic/eco-labelled food (9%), consumption of carbon -labelled food (7%) and consumption of food of local origin (7%). Most interventions that assessed a direct environmental effect measured GHG emissions 10% (58, N = 547), followed by water use (2%) and land use (1%).

Studies commonly evaluated WTP for consumption of different types of labelled foods and animal-source foods. WTP for organic or eco-labelled food constituted 9% of total measured outcomes (49 studies, N = 547). WTP for locally produced foods (24 cases) and carbon-labelled foods (23 cases) were also measured for many interventions and constituted 4% of measured outcomes (N = 547).

#### Knowledge clusters and gaps

**Fig. 9:**
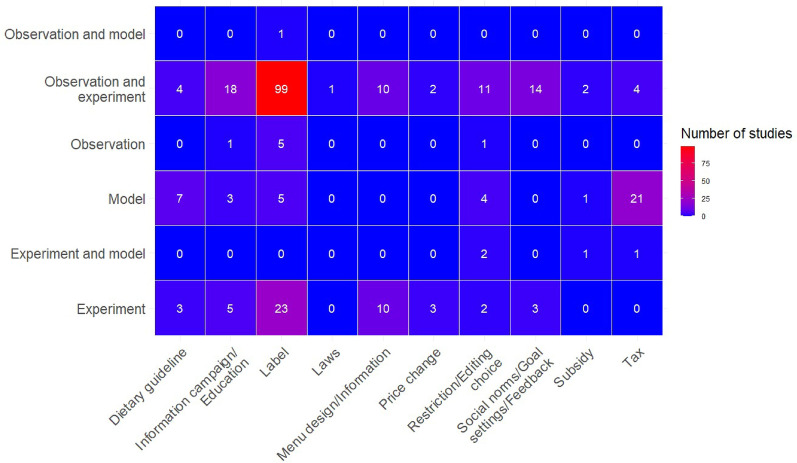
Number of studies per intervention subcategory and study type in the evidence base

A heatmap of the types of interventions across study types revealed clear clusters of experimental and observational studies (99) and experimental studies (23) that evaluated labels (Fig. 9). It also revealed a cluster of studies modelling the effect of taxes (21 studies) and one cluster that evaluated information campaigns/education by observations and experiments (18 studies). On the other hand, few studies used experimental and observational approaches for assessing the effects of, e.g., economic interventions; taxes, subsidies, and price changes (ranging from 0 to 4 studies) (Fig. 9).

**Fig. 10:**
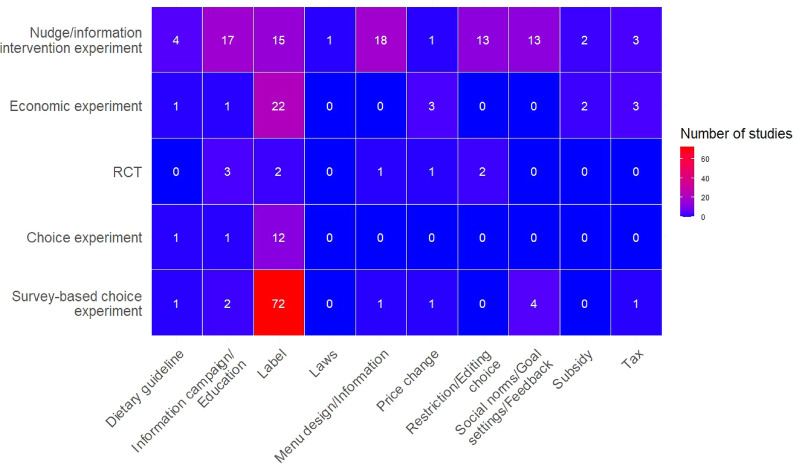
Number of studies per intervention subcategory and experiment types in the evidence base

On comparing the different types of experiments used, we found a large cluster of survey-based choice experiments that evaluated labels (72 studies), as illustrated in Fig. 10. There were also a number of smaller clusters of economic experiments (22 studies), e.g. experimental auctions, regular choice experiments (12 studies) and nudge/information intervention experiments studying labels (15 studies). In addition, there was a cluster of information-based nudge/information intervention experiments studying information campaigns/education (17 studies), menu design/information (18 studies), restriction/editing choice (13 studies) and interventions focusing on social norms/goal setting/feedback (13 studies). There were also evident knowledge gaps, with market-based interventions, dietary guidelines, and laws and regulations seldom tested in experiment-based studies (ranging from 0 to 3 studies) (Fig. 10).

**Fig. 11:**
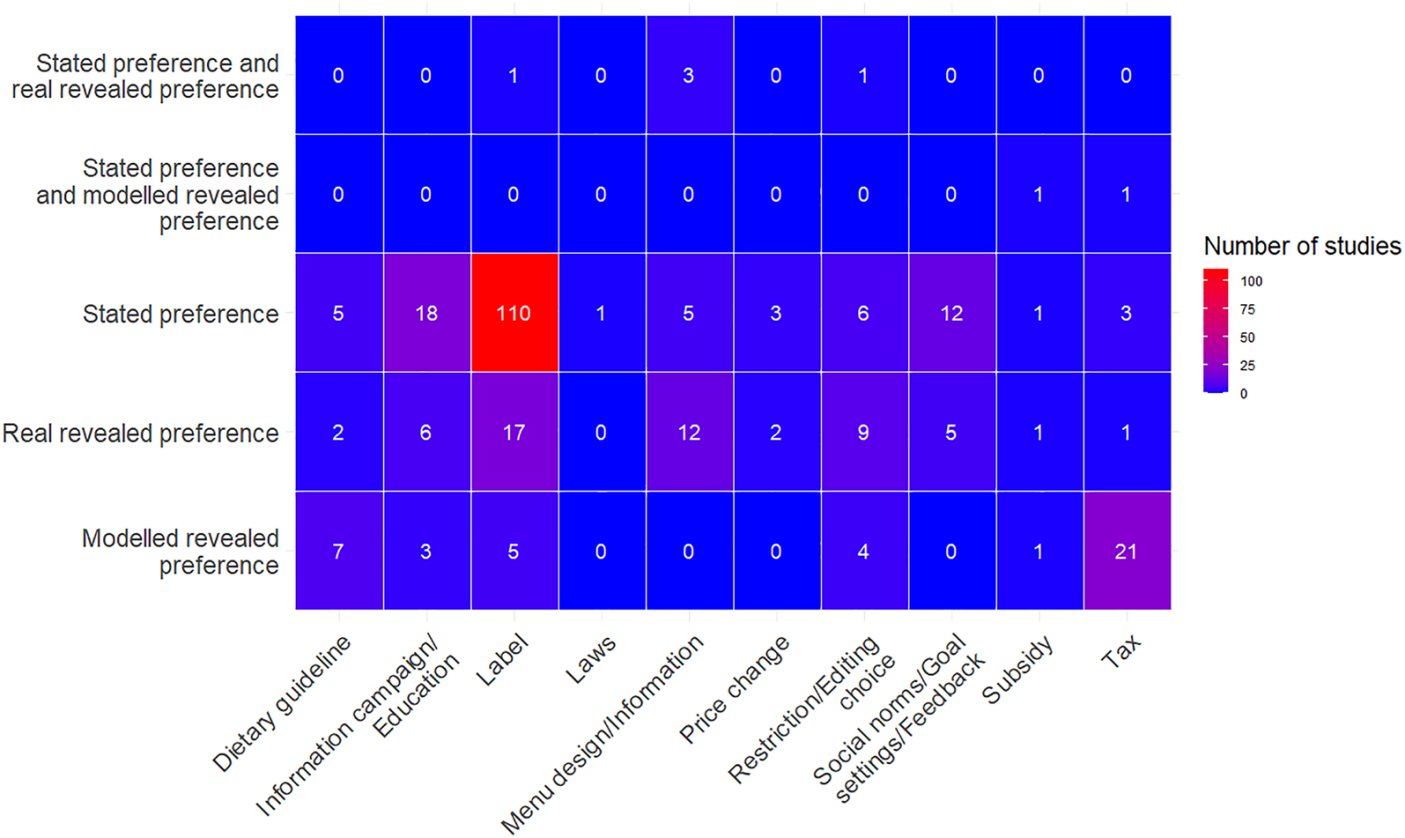
Number of studies per intervention subcategory and type of preference revealed in the evidence base

On looking at type of preference evaluated, we found a large knowledge cluster of studies that evaluated labels by stated preference (110 studies) (Fig. 11). There was also a cluster of studies that measured labels by real revealed preference (17 studies), but it was substantially smaller than that for stated preference. Other small evidence clusters of studies evaluated taxes by modelled revealed preference (21 studies) and information campaigns/education (18 studies) and social norms/goal setting and feedback interventions (12 studies) by stated preference. There were knowledge-gaps with regard to studies on market-based interventions with both stated and real revealed preference (ranging from 0–1 studies) (Fig. 11).

**Fig. 12:**
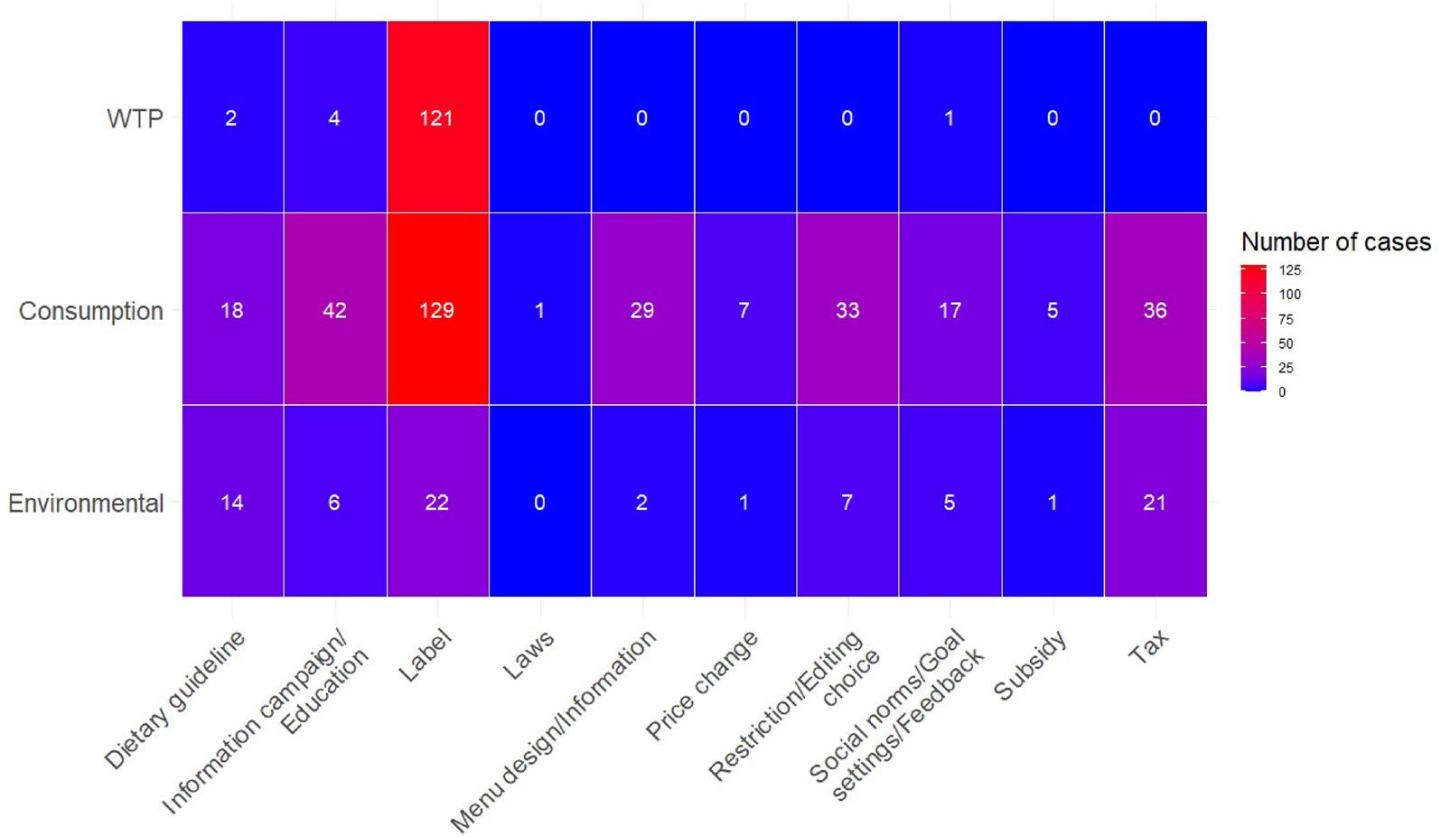
Number of cases of interventions per intervention subcategory and measured outcome in the evidence base

Considering the measured outcomes for interventions, as illustrated in Fig. 12, there were large clusters of cases where labels were by WTP for labelled products (121 cases) and consumption of labelled products (129 cases). A substantially smaller cluster evaluated the direct impact on the environment via labels (27 cases). It was also relatively common to evaluate the effect of taxes directly on the environment (21 cases), although more commonly via consumption of foods (36 cases), or to use consumption of foods as a proxy for evaluating the effect on the environment of restricting/editing choice (33 cases), information campaign/educations (42 cases), and menu design/information (29 cases). Knowledge gaps emerged for evaluating any intervention except labels with WTP. There were also knowledge gaps regarding evaluating information provided on menus (2 cases), laws and regulations (0 cases), price changes (1 case) and subsidies (1 case) with direct environmental impact.

## Limitations of the map

### Limitations of the mapping process

While this systematic mapping employed a comprehensive search strategy, it is important to acknowledge that omission of relevant literature may have occurred, primarily due to language constraints. Specifically, bibliographic sources were searched using English language terms (although grey literature searches on specialist websites included English and multiple Nordic languages). Future reviews should consider expanding the range of languages, e.g. to include Chinese, French and Spanish, to enhance the inclusiveness of the search and minimise potential language-related bias. The formulation of our eligibility criteria resulted in the vast majority of studies being excluded based on the first eligibility criterion on interventions, which could have been avoided with a less strict exclusion criterion.

Our search string comprised a combination of general and specific terms related to interventions and outcomes. Future searches could benefit from inclusion of even more specific terms that emerged from the evidence base mapped in this review, which could be helpful in capturing a broader spectrum of relevant studies. We identified a large number of articles with our search string, including a large quantity of non-relevant articles. Our search string was developed to cover both broad and more detailed search terms, in order to capture as many relevant studies as possible. Acknowledging the diverse nature of the subject and the terminology employed and applied to study and discuss sustainability, we recognise that our coverage of pertinent literature was not exhaustive. For example, when searching the bibliographies of relevant reviews for eligible articles we identified a number of relevant articles to include that were not captured by our bibliographic search, primarily on willingness-to-pay and labelling of food products. This might indicate that there may be more relevant studies on willingness-to-pay and labelling that could be captured with more specific searches. Nevertheless, with the outlined methodology, including listed benchmark studies and stakeholder and expert consultation, we believe that the search string developed successfully covered literature broadly across the subject, enabling thorough mapping of the evidence base.

### Limitations of the evidence base

The evidence base was heavily biased towards high-income countries, and primarily countries such as the United States and United Kingdom (14 and 15% respectively, N = 298). This is not surprising, given our search for literature was predominantly performed in English (see ‘Limitations of the mapping process’ above). Most intervention evaluations were designed as control-impact (52%) while only 13% were designed as before-after-control-impact studies. About 6% of intervention evaluations only had after comparison. This was expected considering that most experiments were survey-based choice experiments. However, it also indicates that evaluation study design in many of the studies could not control for all confounders (by e.g. not including an appropriate control group). Future systematic reviews should conduct careful and thorough critical appraisal of study validity.

## Conclusions

### Implications for policy/management

In systematic mapping of the literature, we identified a large body of evidence on potential public policy interventions that could contribute to influencing food consumption. Restricting our mapping to studies that report effects (measured or estimated) of sustainable consumption policies, we identified 267 unique studies on interventions. Among these, the primary research evidence clustered around the following areas:

1. Labels (N = 133), including organic or eco-labelling (N = 54), carbon labelling (N = 47) and labelling for origin of food (N = 31).
2. Information campaigns/education (N = 27)
3. Menu design/menu information changes (N = 20)
4. Restriction/editing choice/choice context (N = 20).
5. Taxes for more sustainable food consumption (N = 24).

These results reinforce the conclusions of Ammann et al. [26], that the literature on policies for sustainable food consumption is dominated by studies on less intrusive policy instruments, such as labels, information campaigns, changes to menu design and editing the choice context. This is likely because less intrusive interventions are easier to implement and test, as well as likely being more readily accepted by consumers (cf. [45]). Consequently, previous systematic reviews have synthesised the empirical evidence on the effectiveness on such interventions; e.g., Rondoni et al. (2021) reviewed the evidence on labelling [46], while Lehner et al. (2016), Byerly et al. (2018), and Meier et al. (2022) reviewed the evidence on nudging interventions, such as changes to menu design and the choice context [47–49]. However, to our knowledge there has been no previous synthesis of the evidence for taxes for sustainable food consumption. Our systematic mapping also included a number of studies on dietary guidelines adjusted in order to account for the environmental sustainability of diets. Such guidelines were evaluated at national level, but also for public procurement in those studies. However, we excluded a large body of literature modelling scenarios of how a dietary shift can affect the environment, since they did not focus on policy interventions to change food consumption. Future systematic reviews should seek to better identify how diets can be used to stimulate more environmentally sustainable food consumption and would thus include the literature on dietary scenarios and dietary guidelines more broadly.

While we identified substantial evidence in specific areas in this systematic mapping, decision-makers need to be careful in interpreting the evidence, for two main reasons. First, few studies assess intervention effects in real-life settings and instead rely on stated preferences as expressed in, e.g., surveys, laboratory experiments, or models. The predominance of stated or modelled preference studies, in particular, undermines the possibility to draw reliable conclusions about intervention effectiveness. This is due to the intention-behaviour gap, where stated intentions typically overestimate actual behavioural effects [50]. There is a distinction between expressing willingness to alter one’s behaviour, such as pledging to make more environmentally friendly food choices when provided with transparent and thorough information about their environmental impact, and effectively enacting that behavioural change. Our grey literature search did not yield any additional studies testing the impacts of interventions for more sustainable food consumption in real-life contexts. Second, the majority of studies identified in our systematic mapping reported effects in terms of proxies of environmental outcomes, such as consumption or willingness to pay for animal- or plant-based foods or sustainability-certified foods. Only a few studies reported actual environmental outcomes, such as GHG emissions or impact on land and water resources. This is not problematic in cases where consumption and environmental impacts are unequivocally linked, e.g., when using red meat consumption as a proxy for climate impact. Results should be interpreted with more caution, however, when consumption and environmental impacts are not directly linked, e.g., when certified foods or local food consumption are used as proxies for impacts on climate or biodiversity.

Thus, there is a strong need for research on sustainable food policies to ‘step out of the lab’ and enter the real world. This is particularly true for interventions that cannot be assessed in a laboratory or survey setting, such as administrative policies, which are therefore notably absent in our evidence base, or more comprehensive market-based policies. Real-world research will require support and cooperation from public and private sector stakeholders. Ideally, real-world policy should be implemented in ways that enable monitoring, impact evaluation and learning, and implementers of interventions should work in collaboration with researchers to jointly define intervention objectives and testable hypotheses, and facilitate data collection (cf. [51]).

### Implications for research

Research on policy interventions for more sustainable food consumption needs to move from the lab (or the online survey) into the real world and test a wider variety of possible interventions (and combinations of these), such as environmental information campaigns, taxes, regulations and large-scale choice editing. There are methodological challenges in doing so, however. In their call for mainstreaming of impact evaluations of nature conservation, Baylis et al. [51] list a number of reasons for why it is difficult to conduct impact evaluation studies in that area, many of which also apply to policy for more sustainable food consumption: For example, sustainability in food consumption is multi-dimensional and complex and lacks a consensual definition [26, 31], which can present challenges when measuring outcomes. In addition, many contextual and behavioural factors affect the food consumption choices of individuals, making identification of confounding factors difficult, and randomisation is a challenge if interventions are tested at larger scales (in particular for public policy such as dietary guidelines, restrictions or administrative policies). Finally, impact evaluation of large-scale interventions requires scaling-up of available research funding and much stronger collaboration between researchers from different disciplines to assess policy mechanisms, behavioural and economic spillover effects, and outcomes across different dimensions, and between researchers and stakeholders in designing interventions and accompanying impact evaluation plans [10, 51]. This will likely require a mixed methods approach as reflected also in debates in other science-policy fields, such as development or health, on how to combine evidence from ‘gold standard’, randomised control trials and observational studies (see, e.g., [52–54]). In our evidence base, only 41 of 267 studies applied a mixed methods approach. As context is likely to impact intervention effectiveness, it is also important that future research extends its range beyond Europe and North America, to provide evidence on how to counteract the increasing environmental pressures from food consumption across the globe.

## Supplementary Information

**Additional file 1.** Roses form

**Additional file 2.** Academic and grey literature searches

**Additional file 3.** Excluded and non-retrievable articles

**Additional file 4.** Code book

**Additional file 5.** Map database

## Data Availability

The dataset generated and/or analysed in this systematic mapping study is available in the Additional file 5 and at this link. The geographical map of the studies included in the database (with geographical data) can be found here.
