# Supplementary material for: Effects of public policy interventions for environmentally sustainable food consumption: a systematic map of available evidence
Source: Environ Evid. 2024 Apr 12;13:10. doi: 10.1186/s13750-024-00333-6 (PMC11378856; doi:10.1186/s13750-024-00333-6)
Supplement: Supplementary file 1 — Additional file 1. Roses form [file 13750_2024_333_MOESM1_ESM.pdf]

## Additional file 1. ROSES for Systematic Map Reports. Version 1.0

November 2017

Neal R Haddaway, Biljana Macura, Paul Whaley, and Andrew Pullin

**Column descriptions**

Section/sub-section - The sections or sub-sections listed below correspond to sections in CEE systematic review and map protocols and final reports: please see the relevant text under these subheadings for information within a specific review.

Topic - the section or sub-section is further broken down for the purposes of clarity.

Description/Further explanation - Details of what is needed in each section or sub-section are provided, along with practical advice and links to relevant guidance.

Checklist/Meta-data - Checklist items MUST be completed. Meta-data items correspond to information that should be reported as data or short descriptions that will be used to populate the Summary Record and Flow Diagram. Any meta-data items where stages were not performed (e.g. grey literature searching), this should be stated (e.g. 'Not performed').

Author response - authors should select or write a response for meta-data, or select 'yes' or 'no' for checklist items. Further details may be provided in Comments (see below).

Comments - authors may supplement their response with a brief comment.

*Please cite as: Haddaway NR, Macura B, Whaley P, and Pullin AS. 2017. ROSES for Systematic Map Reports. Version 1.0. DOI: 10.6084/m9.figshare.5897299.*

| Item number | Section/sub-section | Topic | Description | Further explanation | Checklist/meta-data | Author response | Comments |
|---|---|---|---|---|---|---|---|
| 1 | Title | Title | The title must indicate that it is a systematic map, and should indicate if it is an update/amendment: e.g. "…A systematic map update." | The title should normally be the same or very similar to the review question. | Meta-data | Effects of public policy interventions for environmentally sustainable food consumption: a systematic map of available evidence | |
| 2 | Type of review | Type of review | Select one of the following types of review: systematic map, systematic map update, systematic map amendment | See CEE Guidance on systematic mapping [1], and on amendments and updates [2] | Meta-data | Systematic map | |
| 3 | Authors' contacts | Authors' contacts | The full names, institutional addresses and email addresses for all authors must be provided. | | Checklist | Yes | |
| 4 | Abstract | Structured summary | The abstract of the manuscript must not exceed 500 words and must be structured into separate sections: Background, the context and purpose of the review, including the review question; Methods, how the review was performed (specifically mention search strategy, inclusion criteria, critical appraisal (optional), meta-data extraction and coding, and narrative synthesis); Results, the main findings, including results of search and assessment of evidence base; Conclusions, brief summary and potential implications for policy/management and research. | | Checklist | Yes | |
| 5 | Background | Background | Describe the rationale for the review in the context of what is already known. Reviews must indicate why this study was necessary and what it aims to contribute to the field. | A theory of change and/or conceptual model should be presented that links the intervention or exposure to the outcome. | Checklist | Yes | |
| 6 | Stakeholder engagement | Stakeholder engagement | The actual role of stakeholders throughout the review process (e.g. in the formulation of the question) must be described and explained (using a broad definition of 'stakeholder', including e.g. researchers, funders and other decision-makers; see [3]) | | Checklist | Yes | |
| 7 | Objective of the review | Objective | Describe the primary question and secondary questions (when applicable). | The primary question is the main question of the review. The secondary questions are usually linked to sources of heterogeneity (effect modifiers). | Checklist | Yes | |
| 8 | | Definition of the question components | Provide reference to the question key elements, e.g. population(s), intervention(s)/exposure(s), comparator(s), and outcome(s). | For other question types see [4,5] | Meta-data | Population: Private and public food consumption in any geographic or economic setting. Intervention: Public policy interventions with the explicit aim to achieve more environmentally sustainable food consumption patterns (implemented by national or sub-national governments or suggested). This includes policy interventions aimed at achieving change in consumption of e.g., meat, plant-based foods, deforestation-prone foods, or certified products. The intervention should have the end goal to directly or indirectly influence consumer choice towards more sustainable foods. Outcome: Anticipated or actual change in any type of environmental impacts of food production, or proxies thereof including e.g., change in meat consumption, plant-based foods, consumption of deforestation prone foods or certified products. Studies that measure and/or discuss health outcomes only will not be included, although the interventions included in such studies, e.g., reduced overconsumption or consumption of red meat, might lead to environmental gains. Studies that do not explicitly measure environmental outcomes (directly or indirectly) will not be included. | |
| 9 | Methods | Protocol | Provide citation, DOI or open-access link to published protocol. | The protocol should be peer-reviewed and publicly available online (open access). | Meta-data | https://doi.org/10.1186/s13750-022-00271-1 | |
| 10 | | Deviations from protocol | Describe any ways in which the final methods of the review deviate from those set out in the protocol along with a justification. | | Checklist | Yes | |
| 11 | Searches | Search strategy | Detail the search strategy used, including: database names accessed, dates of searching, institutional subscriptions (or date ranges subscribed for each database), search options (e.g. 'topic words' or 'full text' search facility), efforts to source grey literature, other sources of evidence (e.g. hand searching, calls for evidence/submission of evidence by stakeholders). | | Checklist | Yes | |
| 12 | | Search string | Provide Boolean-style full search string and state the platform for which the string is formatted (e.g. Web of Science format) | | Meta-data | Web of Science: Topic: (((food or meal* or diet or eating) NEAR/2 (purchas* or select* or choice* or reduc* or choose or decision or consum* or intake or behav* or habit*)) or "product select*" or "food products" or menu or "food environment" or "dietary pattern*" or catering or ((beverage* or grocery or groceries or fish or seafood or beef or meat or dairy or milk or vegetable* or legume* or "meat alternative" or "organic food" or "local food") NEAR/2 (consum* or choice* or choose or select* or market or demand* OR reduc*))) AND (policy or policies or legislat* or law* or ((label* or labl* or certifi*) NEAR/2 (food or ecol* or sustainab* or carbon or climate)) or ecolabl* or ecolabel* or eco-labl* or eco-label* or eco-certif* or guideline* or guidance or incenti* or intervent* or nudg* or subsid* or stimul* or persua* or "voluntary agreement*" or roundtable* or forc* or innovat* or directive* or regulation or regulations or education or ((plate* or serving-size or "serving size") NEAR/1 ration*) or ((carbon or consum* or output or environmental) NEAR/2 (tax* or information or standard* or ban* or prohibit* or limit* or sanction*)) or "green criteria" or "green public procurement" or "public procurement") AND ("climate change" or "climatic change" or "global warming" or "greenhouse gases" or ghg or "greenhouse effect" or "greenhouse gas" or "carbon emission*" or "carbon footprint" or "water footprint" or "land use" or "biodiver*loss*" or ecosystem or overfishing or pollution or "over fishing" or deforest* or (reduction* NEAR/2 emission*) or (environment* NEAR/2 (impact* or consequence* or assess* or evaluat* or indicator* or mitigat*)) or "plant based food" or "plant-based food" or "planetary health diet" or plant-forward or "pro-environmental" or "local food" or "seasonal food" or "eat less" or overconsumption or overeating or flexitarian or vegan or vegetarian or pescetarian or "meat reduction" or "beef reduction" or (sustainab* NEAR/2 (consum* or diet* or food or fisher*))) | |
| 13 | | Languages - bibliographic databases | List languages used in bibliographic database searches | | Meta-data | English | |
| 14 | | Languages – grey literature | List languages used in organisational website searches and web-based search engines | | Meta-data | English, Swedish, Norwegian, Danish | |
| 15 | | Bibliographic databases | Provide the number of bibliographic databases searched | | Meta-data | 5 | |
| 16 | | Web-based search engines | Provide the number of web-based search engines searched | | Meta-data | 1 | |
| 17 | | Organisational websites | Provide the number of organisational websites searched | | Meta-data | 28 | |
| 18 | | Estimating comprehensiveness of the search | Describe the process by which the comprehensiveness of the search strategy was assessed (i.e. list of benchmark articles) | | Checklist | Yes | |
| 19 | | Search update | Describe any update to searches undertaken during the conduct of the review | Compulsory (if update performed). A search update is good practice if original searches were performed more than two years prior to review completion. | Checklist | No | |
| 20 | Article screening and study inclusion criteria | Screening strategy | Describe the methodology for screening articles/studies for relevance. Methods for consistency of screening decisions (at title, abstract, and full texts levels) checking must be described. | | Checklist | Yes | |
| 21 | | Inclusion criteria | Describe the inclusion criteria used to assess relevance of identified articles/studies. These must be broken down into the question key elements (e.g. relevant subject(s), intervention(s)/exposure(s), comparator(s), outcome(s), study design(s)) and any other restrictions (e.g. date ranges or languages). | | Checklist | Yes | |
| 22 | Critical appraisal | Critical appraisal strategy | Describe here the method used for critical appraisal of study validity (including assessment of individual studies and the evidence base as a whole). Describe how repeatability of critical appraisal of study validity was tested. | Optional | Checklist | No | |
| 23 | | Critical appraisal used in synthesis | Describe how the information from critical appraisal was used in synthesis. | Compulsory if critical appraisal performed | Checklist | n/a | |
| 24 | Meta-data extraction and coding strategy | Meta-data extraction and coding strategy | Describe the method for meta-data extraction and coding for studies, providing lists of variables that will be extracted as meta-data and those that will be coded. Describe how repeatability of meta-data/data extraction and coding was tested. | | Checklist | Yes | |
| 25 | | Approaches to missing data | Describe any process for obtaining and confirming missing or unclear information or data from authors. | | Checklist | Yes | |
| 26 | Data synthesis and presentation | Narrative synthesis strategy | Describe methods used for narratively synthesising the evidence base in the form of descriptive statistics, tables (including SM database) and figures. | | Checklist | No | |
| 27 | | Knowledge gap and cluster identification strategy | Describe the methods used to identify and/or prioritise key knowledge gaps (unrepresented or underrepresented subtopics that warrant further primary research) and knowledge clusters (well-represented subtopics that are amenable to full synthesis via systematic review). | | Checklist | Yes | |
| 28 | | Demonstrating procedural independence | Describe the role of systematic reviewers (who have also authored articles to be considered within the review) in decisions regarding inclusion or critical appraisal of their own work. | Reviewers who have authored articles to be considered within the review should be prevented from unduly influencing inclusion decisions, for example by delegating tasks appropriately. | Checklist | Yes | |
| 29 | Results (review findings) | Description of review process | Describe the review process including the volume of evidence identified from all sources and retained through each stage of the review. Must also display the number of articles/studies included at all stages of the review in a flow diagram, including the number of articles/studies excluded at each stage. | | Checklist | Yes | |
| 30 | | Number of search results | Provide the number of search results from bibliographic databases (including updates if conducted) prior to duplicate removal. | This number should not include web-based search engine or organisational website searches: this will help assessment of the efficiency of the primary search string. | Meta-data | 27819 | |
| 31 | | Number of search results after duplicate removal | Provide the total number of search results from bibliographic database searches following duplicate removal. | This number should not include web-based search engine or organisational website searches: this will help assessment of the efficiency of the primary search string. | Meta-data | 19254 | |
| 32 | | Full text screening excludes | Additional file containing list of and reasons for full text exclusions. | | Checklist | Yes | |
| 33 | | Title screening results | Provide the number of articles retained following title screening. | Optional if screening titles and abstracts together | Meta-data | NA | |
| 34 | | Abstract screening results | Provide the number of articles retained following abstract screening. | Optional if screening titles and abstracts together | Meta-data | NA | |
| 35 | | Title and abstract screening results | Provide the number of articles retained following title and abstract screening. | Optional if screening titles and abstracts separately | Meta-data | 1605 | |
| 36 | | Retrieval results | Provide the number of articles retrieved at full text. | | Meta-data | 1545 | |
| 37 | | Unobtainable articles | Additional file containing list of unobtainable articles. | | Checklist | Yes | |
| 38 | | Full text screening results | Provide the number of articles retained following full text screening. | | Meta-data | 227 | |
| 39 | | Consistency checking: screening | Results of consistency checking at all stages (screening, meta-data extraction and coding, critical appraisal) must be provided. Provide the number of titles, abstracts and full texts screened and checked for consistency by two or more reviewers as a fraction of the total (e.g. Title: 2000/20000; Abstract: 500/5000: Full text: 10/100). | | Checklist | Yes | Title/Abstract: 474/19254; Full text: 110/1545; Meta-data extraction 20/227 |

| | | | | | | |
|---|---|---|---|---|---|---|
| 40 | | Narrative synthesis | Describe the body of evidence identified using figures and tables, avoiding vote-counting (tallying of studies based on results; direction or significance). Each must be presented with descriptive information (meta-data). Describe the validity of individual studies and the evidence base as a whole (if critical appraisal conducted). | | Checklist | Yes |
| 41 | | Systematic map database | Additional file containing meta-data and coding for included studies. | | Checklist | Yes |
| 42 | | Limitations of the review | Discuss possible limitations in the methods used. | | Checklist | Yes |
| 43 | | Limitations of the evidence base | Discuss possible limitations in the evidence base. | | Checklist | Yes |
| 44 | Conclusions | Knowledge gaps and clusters | Describe knowledge gaps (unrepresented or underrepresented subtopics that warrant further primary research) and knowledge clusters (well-represented subtopics that are amenable to full synthesis via systematic review) | | Checklist | Yes |
| 45 | | Implications for policy/management | Summarise the state of the evidence base and discuss the way in which the identified evidence may inform policy/practice decision making in relation to the review/map question. | Reviews must not include practical environmental management recommendations or advocacy. | Checklist | Yes |
| 46 | | Implications for research | Discuss the way in which the identified evidence may inform research including options for increasing the reliability of study design that could improve future research. | In this section some advocacy for future research on the reviewed topic is permissible provided it is clearly justified by the review outcome/critical appraisal of study validity. | Checklist | Yes |
| 47 | Declarations | Competing interests | Describe of any financial or non-financial competing interests that the review authors may have. | | Checklist | Yes |

**References**

[1] James, K.L., Randall, N.P. and Haddaway, N.R., 2016. A methodology for systematic mapping in environmental sciences. Environmental Evidence, 5(1), p.7.
[2] Bayliss, H.R., Haddaway, N.R., Eales, J., Frampton, G.K. and James, K.L., 2016. Updating and amending systematic reviews and systematic maps in environmental management. Environmental Evidence, 5(1), p.20.
[3] Haddaway, N.R., Kohl, C., da Silva, N.R., Schiemann, J., Spök, A., Stewart, R., Sweet, J.B. and Wilhelm, R., 2017. A framework for stakeholder engagement during systematic reviews and maps in environmental management. *Environmental Evidence* , *6* (1), p.11.
[4] Collaboration for Environmental Evidence. 2018. Guidelines and Standards for Evidence synthesis in Environmental Management. Version 5.0. www.environmentalevidence.org/information-for-authors.
[5] Leeds Institute of Health Sciences. https://medhealth.leeds.ac.uk/info/639/information_specialists/1500/search_concept_tools. Accessed 12/11/2017.
